# Do discontinuities in marginal reimbursement affect inpatient psychiatric care in Germany?

**DOI:** 10.1007/s10198-020-01241-5

**Published:** 2020-11-09

**Authors:** Clara Pott, Tom Stargardt, Udo Schneider, Simon Frey

**Affiliations:** 1 Hamburg Center for Health Economics (HCHE), University of Hamburg, Esplanade 36, 20354 Hamburg, Germany; 2 Techniker Krankenkasse, Bramfelder Straße 140, 22305 Hamburg, Germany

**Keywords:** Health care financing, Prospective payment system, Length of stay, Marginal payment incentives, Hospital behaviour, Mental health care, Analysis of health care markets (I11), Government policy, Regulation, Public health (I18)

## Abstract

This paper examines the behaviour of mental health care providers in response to marginal payment incentives induced by a discontinuous per diem reimbursement schedule with varying tariff rates over the length of stay. The analyses use administrative data on 12,627 cases treated in 82 psychiatric hospitals and wards in Germany. We investigate whether substantial reductions in marginal reimbursement per inpatient day led to strategic discharge behaviour once a certain length of stay threshold is exceeded. The data do not show gaps and bunches at the duration of treatment when marginal reimbursement decreases. Using logistic regression models, we find that providers did not react to discontinuities in marginal reimbursement by significantly reducing inpatient length of stay around the threshold. These findings are robust in terms of different model specifications and subsamples. The results indicate that if regulators aim to set incentives to decrease LOS, this might not be achieved by cuts in reimbursement over LOS.

**Electronic supplementary material:**

The online version of this article (10.1007/s10198-020-01241-5) contains supplementary material, which is available to authorized users.

## Introduction

In response to soaring health care expenditures, prospective payment systems (PPS) in inpatient care have been implemented in almost every advanced health care system worldwide. With PPS, payment rates are determined ex ante, and there is no link to quality, cost-reducing effort, quantity of services devoted to each patient’s treatment or unit costs of an individual health care provider [1, 2]. PPS typically require some type of classification systems (e.g. diagnosis-related groups), where inpatient cases are assigned to medically distinguishable groups that are intended to be homogenous in terms of resource utilization for treatment [3]. Payment rates for each group ought to reflect the average costs of providing care.

An advantage of such systems is that they set incentives to prohibit over-provision and to minimize costs of care, as additional treatment would increase hospital costs without generating additional profit [2]. However, PPS may also cause unintended hospital behaviour that is unfavourable to quality of care because providers may base decisions not only on medical benefit considerations, but also on maximizing reimbursement given the financial incentives of the payment system [4]. Ellis and McGuire [5] model this decision process based on the assumption that physicians serve as agents who are interested in hospital profits and patient benefit at the same time. They show that if physicians undervalue patient benefit relative to hospital profit, PPS may result in under provision of services, e.g. reductions in inpatient length of stay (LOS).

There is evidence that providers of health care respond to financial incentives (see McGuire [6] for an overview). Concerning the hospital sector, several studies examined supplier responses following a switch from cost-based reimbursement to prospective payment (see Cutler and Zeckhauser [7] for a review). Evidence suggests that LOS is significantly reduced following the introduction of prospective payment [8–10]. However, most of the literature investigates the introduction of a per-case payment system, where hospitals lose marginal reimbursement for providing additional treatment [11]. Consequently, evaluations of the effect of marginal reimbursement incentives on treatment duration are challenging and scarce.

Recently, the introduction of per diem prospective payment systems has become topical because the reform of mental health care financing to improve cost-efficiency is currently of public concern. Because uncertainty and variations in treatment are likely to be high in the mental health care market [12], countries such as the US (under Medicare), Switzerland and Germany decided to base PPS for mental health-care providers on per diem instead of per-case prospective payments (see Mason and Goddard [13] and Schneeberger et al. [14] for descriptions of respective payment systems). Those systems are prospective in terms of the quantity and intensity of services provided within each day, but retrospective with respect to LOS.

As per diem payments have fewer incentives for cost containment [15], declining rates of payment may be used to disincentivize excessively long hospital stays [16]. If per diem rates, i.e. hospitals’ total revenue for an inpatient day, exceed marginal costs, a positive marginal contribution is realized and there exists an inherent incentive to expand LOS. In contrast, if marginal reimbursement rates are lower than marginal costs, hospitals incur losses and thus may have an incentive to reduce treatment days. Varying per diem rates over LOS provide an opportunity to examine whether hospitals respond to marginal reimbursement incentives by manipulating patients’ LOS.

Several studies address reimbursement incentives induced by discontinuously per diem prospective payment. Using data on inpatient psychiatric patients in Switzerland, Pletscher [17] examined whether the introduction of a mixed reimbursement system led to changes in the timing of discharge. The reimbursement scheme consisted of relatively high per diem rates up to day 5, a per case payment on day 6 and comparably lower per diem rates for all subsequent days of treatment. The author hypothesized that the decrease in marginal revenue on day 6 increases the probability of discharge. His results provide evidence in favour of a marginal price effect on LOS in inpatient psychiatric facilities. Conducting a subgroup analysis, he shows that a significant change in the hazard curve is found for patients for whom only the mixed payment tariff is applied. Patients for whom mixed tariffs and government contributions in the form of retrospective annual payments based on accumulated losses were reimbursed, no significant effect could be detected. Pletscher [17] concludes that the reduction in marginal revenue must be sufficiently large to establish incentives to reduce LOS.

Increasing discontinuous reimbursement schedules and their impact on treatment duration and LOS have also been subject to analysis. Douven et al. [18] evaluate the introduction of a discontinuous reimbursement schedule for self-employed mental health-care providers in the Netherlands. They find that providers respond to price incentives by treating patients longer to surpass a treatment duration threshold. In doing so, they can almost double revenues (e.g. for the specialty depression).

Two recently published works of Eliason et al. [19] and Einav et al. [20] examine the impact of the introduction of a discontinuous prospective reimbursement schedule for long-term care hospitals in the USA [19, 20]. Hospitals are reimbursed a daily amount of approximately $1300 on average until a threshold LOS is crossed. Then, they receive a large lump sum payment of approximately $ 13,500 for keeping a patient an additional day, but no reimbursement for all following days [20]. Both studies find that discharges increase substantially once the threshold is passed.

This paper analyses discharge behaviour of mental health care providers in response to marginal payment incentives induced by a discontinuous per diem reimbursement schedule in Germany. We contribute to the existing literature in several ways. (1) In contrast to most previous studies, we do not exploit the introduction of PPS but evaluate a within-system change in marginal reimbursement. This is important because the implementation of a new payment system is usually accompanied by a complete reformation of reimbursement policies, e.g. the revision of accounting regulations and patient classification systems, which makes it difficult to isolate the effect of reimbursement incentives. (2) We investigate PPS-based per diem rates instead of PPS-based case payments, a concept that offers a more refined approach to setting reimbursement incentives. Thus, we exploit a discontinuously declining marginal reimbursement. (3) We do so in a mental health setting, where classification is difficult due to heterogeneity across cases. This specialty has been excluded from most diagnosis-related group (DRG) systems and prospective payment is a relatively new phenomenon in this area. Understanding providers’ reactions to financial incentives in this setting is highly relevant, as many governments are struggling with designing mental health care payment systems for inpatient services.

The remainder of this paper is structured as follows. Section 2 outlines the features of the reimbursement system and describes the regulatory reform that we exploited for analysis. Section 3 offers the specifications of the empirical analysis, Section. 4 summarizes the results of the descriptive analysis and regression estimations, and the discussion about marginal price incentives is presented in Section 5.

## Background

**Fig. 1:**
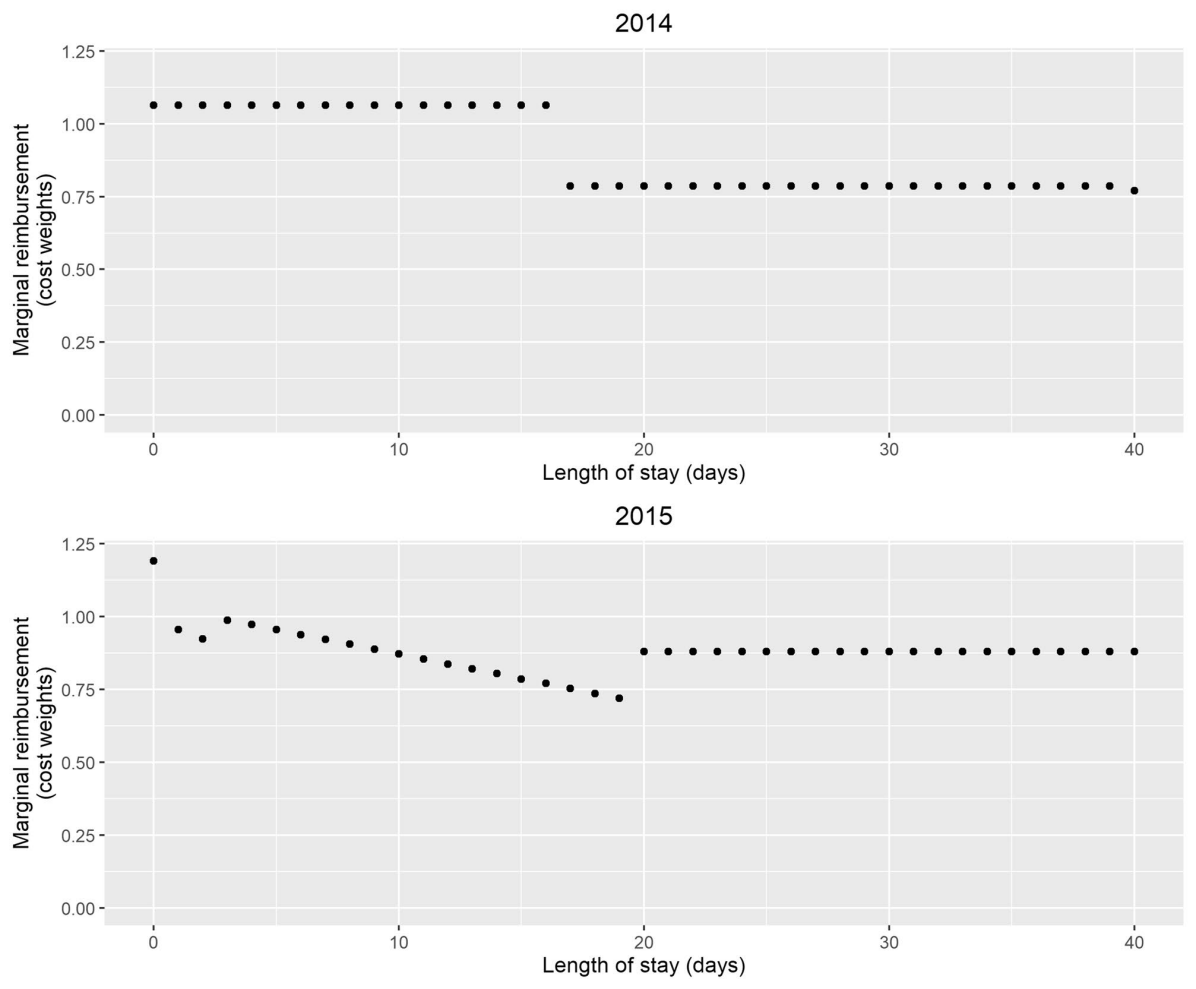
Marginal reimbursement for the PEPP “Affective somatoform or stress disorders, anxiety or sleeping problems, < 65 years, without complicating diagnoses or constellations”

Until 2013, inpatient psychiatric and psychosomatic hospitals and wards in Germany were reimbursed at a department-specific daily rate. To contain costs and align reimbursement more closely to the intensity of resource use in inpatient psychiatric care, the prospective payment system PEPP (“pauschalierendes Entgeltsystem in der Psychiatrie und Psychosomatik”) for psychiatric and psychosomatic inpatient facilities was introduced. Its application was optional between 2013 and 2017 and became obligatory for all hospitals in January 2018. Similar to DRGs, the PEPP system aims to classify patients into cost-homogeneous groups (so-called PEPPs) based on the treating department, type of treatment (inpatient, part residential), age, sex, principal diagnosis, secondary diagnoses and procedures. There are currently 84 PEPPs listed in the national catalogue [21]. PEPP prices are based on cost weights for each day of a hospital stay. Cost weights are normalized such that the average per diem cost weight (Day Mix Index) is equal to 1.0 (see InEK [22] for a detailed explanation of the normalization). An example is displayed in Fig. 1 (left panel), where, i.e. marginal reimbursement for the PEPP “Affective somatoform or stress disorders, anxiety or sleeping problems, < 65 years, with complicating diagnoses or constellations” follows a discontinuous discrete step function.

Total reimbursement is determined by multiplying the sum of cost weights (CW) over total length of stay by a base payment rate (BPR). Hence, total payment (TP) to hospital *h* for a given patient in PEPP *p* with *d* inpatient days of care is determined as follows:

$${\text{TP}}_{p,h,d} = \mathop \sum \limits_{d = 1}^{D} {\text{CW}}_{p,d} \times {\text{BPR}}_{h} .$$

The design of the reimbursement system led to substantial decreases in marginal reimbursement if LOS exceeded a certain threshold *t*, which was determined based on quantiles of the LOS distribution. In the example depicted in Fig. 1 (upper panel), hospitals face a 26% reduction in marginal reimbursement for treating a patient of this cost group after day 17. Those discontinuities in marginal reimbursement apply to all cost groups considered and induce per diem reimbursement reductions of up to 32%. Average reimbursement, i.e. the sum of per diem rates divided by the number of treatment days, is also affected by the cut in per diem rates; however, the decrease is much smaller (− 1.6% at the threshold LOS in the example displayed above).

In response to criticism from several professional associations that the discontinuous reimbursement schedule does provide incentives to reduce the LOS of patients, especially to the detriment of severe cases [23, 24], the self-governing bodies (statutory and private health insurance funds and the German Hospital Federation) decided to avoid abrupt reductions in marginal reimbursement by applying a continuous reimbursement function. As of 2015, reimbursement underwent a reform, i.e. reductions in cost weights were smoothed and the cost weight assigned to the last day of treatment was used for all preceding days of an inpatient stay.

In comparison to the reimbursement in 2014, this led to a marginal reimbursement without abrupt, substantial decreases for longer LOS, as illustrated in Fig. 1 (lower panel). The new design of the PEPP system rather led to an increase in marginal reimbursement if the patients’ LOS exceeded 20 days.

This setting allows us to observe potential behavioural adjustments of hospitals in response to regulatory interventions. More precisely, in this paper, we aim to compare discharge patterns in 2014, where discontinuities in marginal reimbursement applied, to those in 2015, where the reimbursement function was smoothed.

We examine whether economic incentives led to reductions in LOS and whether there has been an abnormal increase in the number of hospital discharges in the period immediately preceding an impending reimbursement reduction.

## Methods

### Data

Analyses of this study are based on administrative data from a large German sickness fund (Techniker Krankenkasse), which provides coverage for 10.5 million people. The dataset contains information on all provided services paid by the sickness fund (i.e. in- and outpatient services, prescription data, medical diagnoses, etc.) and includes longitudinal patient-level information on socio-demographics. Furthermore, information on psychiatric facilities (e.g. affiliated day clinics) were retrieved. To measure additional provider characteristics (e.g. located in rural or urban areas), we merged data from the Federal Institute for Research on Building, Urban Affairs and Spatial Development [25].

First, all inpatient cases with a psychiatric hospital stay reimbursed under the PEPP system between January 2014 and December 2015 were selected from the data. Those cases were divided into an intervention group, i.e. cases treated in 2014 for which discontinuous marginal reimbursement applied and a control group comprising all relevant cases treated in 2015, when abrupt reductions in cost weights were removed.

A period of one year prior to the admission date of each case was monitored to collect information for the calculation of patient-level risk profiles, which we used for risk adjustment in the regression models. Patients who were not insured over the entire individual specific observation period were excluded from the study. As the implementation of PEPP had been a voluntary decision up to 2018, there is a possible selection effect. In other words, treatment may be explained by unobserved hospital characteristics. To counteract this effect, we keep the hospital population constant and eliminate all cases treated in hospitals that did not apply the reimbursement system in both years.

In 2014 and 2015, inpatient cases were assigned to 43 different PEPPs. As we aim to achieve a counterfactual situation, cases in the intervention and control group should exhibit similar characteristics that affect LOS [26]. To enhance comparability, we restricted analysis to PEPPs, whose grouping algorithm did not underlie relevant changes, i.e. in assigned main diagnoses, over the two years such that the cost groups comprise the same types of cases. Furthermore, cases from cost groups without abruptly declining per diem cost weights in 2014 were excluded. We further discard patient suffering from mental and behavioural disorders due to psychoactive substance use as they often discharge themselves prematurely against medical advice [27] such that the decision about the timing of discharge is not made by the treating physician.

Data selection left us with eight cost groups (see Table 1). Some of those cost groups were subject to minor grouping changes, e.g. modifications in assigned secondary diagnoses. For example, patients classified as PEPP PK04 (“Affective, neurotic, stress, somatoform and sleep disorders”) with a secondary diagnosis “bulimia nervosa” were grouped into PK04A in 2014, but PK04B in 2015. We accounted for these changes by eliminating all cases with characteristics that could have led to different classifications in both years.

**Table 1:** Marginal price change in percent

PEPP	Designation^a^	Threshold LOS^b^	Per diem cost weight < threshold LOS	Per diem cost weight ≥ threshold LOS	Marginal price change in %	Observations
PA03A	Schizophrenia, schizotypal, delusional and other psychotic disorders, age > 64 years or severe cases	20	1.1354	0.9509	− 16.25	320
PA03B	Schizophrenia, schizotypal, delusional and other psychotic disorders, age < 65 years	17	1.0682	0.864	− 19.12	2159
PA04A	Affective, neurotic, stress, somatoform and sleep disorders, age > 89 years or severe cases	17	1.22	1.0084	− 17.34	215
PA04B	Affective, neurotic, stress, somatoform and sleep disorders, age > 64 years	20	1.0897	0.8676	− 20.38	1410
PA04C	Affective, neurotic, stress, somatoform and sleep disorders, age < 65 years	17	1.0642	0.787	− 26.05	6584
PA14B	Personality, behavioural, eating and others disorders, age < 65 years	10	1.2463	0.8854	− 28.96	870
PK04B^c^	Affective, neurotic, stress, somatoform and sleep disorders	18	1.9245	1.3127	− 31.79	773
PK10Z^c^	Eating and feeding disorders	22	2.107	1.5383	− 26.99	296

^a^For more detailed information, see Online Appendix

^b^The reported LOS refers to the first day reduced marginal reimbursement being accounted for

^c^PEPPs PK04B and PK10Z comprise cases treated in child and juvenile psychiatry, while the others contain cases of adult psychiatry

Table 1 shows the selected cost groups and displays the first day of inpatient stay, when reduced marginal reimbursement applies along with the relative reduction in marginal reimbursement, which varies between 16.25 and 31.79%. The number of observations per cost group ranges from 215 to 6584. Overall, the final data set includes 12,627 cases for 10,402 individual patients at 82 hospitals.

### Empirical strategy

This study employs a quasi-experimental, retrospective design to identify the causal effect of changes in marginal reimbursement on hospitals’ timing of discharge.

Marginal reimbursement effects are expected to manifest at threshold *t* (see Fig. 1, left panel), where an abrupt reduction in marginal reimbursement applies. Assuming that marginal costs of treatment do not discontinuously change at this point of treatment duration, the relation between marginal revenue and marginal costs substantially changes to the detriment of hospitals. Hence, there may be an incentive to discharge strategically when either marginal reimbursement falls below marginal costs or hospitals immediately recruit a new patient for whom marginal revenues are higher.

We estimated a logistic regression model, where the dependent variable indicates whether a patient was discharged from the hospital within a predefined time period prior to threshold *t*. Two different specifications were constructed to model the probability of being discharged within 3 and 5 days before reduced rates apply. The probability was estimated as a function of the reimbursement change, which is parameterized by a binary variable “Treat” taking on the value of one for admissions under discontinuous marginal reimbursement in 2014. If marginal price incentives affect hospital discharge behaviour, the estimated coefficient is expected to be positive. Additional independent variables are used to control for individual and hospital-specific characteristics. The basic equation is

$$\log \left( {\frac{{P \left( {Y_{i} = 1} \right)}}{{1 - P \left( {Y_{i} = 1} \right)}}} \right) = \alpha + \beta {\text{TREAT}}_{i} + {\gamma^{\prime}{\text{CASE}}_{i}} + {\delta ^{\prime}{\text{HOSP}}_{i}} + \epsilon_{i} ,$$

where ${\text{CASE}}_{{\varvec{i}}} \user2{ }$ is a vector of covariates for case $i$ and ${\text{HOSP}}_{{\varvec{i}}}$ is a vector of hospital-specific covariates. $\alpha$ and $\beta$ are coefficients and $\gamma , \delta$ are vectors of coefficients to estimate. $\epsilon_{i}$ denotes the error term, which is assumed to be independent of the explanatory variables.

The vector ${\text{CASE}}_{{\varvec{i}}} \user2{ }$ comprises several case-specific socio-demographic characteristics and comorbidities that are potential confounders for LOS. Previous studies found sex [28] and age [29] to be determinants of LOS in inpatient mental health care. Evidence further suggests the relationship between duration of hospitalizations for psychiatric inpatients and age to be non-linear [30]. We therefore incorporate sex, age and age^2^ as controls in our regression model. Comorbidities were measured using 31 dichotomous diagnosis-based classification groups [31] and 32 dichotomous drug prescription-based classification groups [32]. Four diagnostic-based groups (Alcohol abuse, Drug abuse, Psychoses, Depression) and four pharmacy-based groups (Depression, Psychotic illness, Bipolar disorder, Anxiety and tension) were excluded because adjusting for them would probably induce post-treatment bias [33, 34].

${\text{HOSP}}_{i}$ is a vector of hospital-specific controls that may affect the timing of discharge. As the literature suggests that adjustment to new payment incentives is time delayed [35], we included a variable indicating the number of days PEPP has been implemented. A binary indicating whether a hospital is located in an urban area (> 20,000 inhabitants) was incorporated, as facilities in urban and rural regions exhibit structural differences which may influence their treatment behaviour [36]. Further, we added a dichotomous variable indicating whether a patient was treated in a small hospital (< 100 cases in the observation period), as hospital size was found to be a determinant of LOS [37]. If a hospital has the option of admitting patients to an ambulatory facility or day clinic, the probability for early discharges may increase [38]. This is taken into account by adding binary controls indicating whether a psychiatric outpatient or day clinic is affiliated with the hospital.

If hospitals alter their discharge behaviour in response to reimbursement incentives, we suspect them to shift discharges for those patients whose LOS only just exceeds the threshold of interest. This can be expected because hospitals (if they do at all) are likely to manipulate LOS in a way that will minimize expected medical/health consequences for the patients [5]. Premature discharges would least harm those patients who would have been discharged a few days later anyway. In addition to estimating with the full sample, we estimate the regression model for two subsamples restricted to cases that are discharged either 3 (5) days before versus after threshold t.

### Sensitivity analysis and robustness checks

First, we test the sensitivity of the regression results by estimating an alternative model specification with hospital fixed effects. We aim to control for unobserved hospital effects that influence discharge behaviour and are not captured in the hospital characteristic controls employed in the basic regression model. We chose a specification with hospital fixed instead of random effects, as hospital random effects would be correlated with other explanatory variables (see Norton et al. [39]).

We further check the robustness of our results by estimating the basic regression model on two subgroups. For the first subgroup analysis, we exclude all cases discharged from hospitals in December or admitted to the hospital in January to eliminate potential accounting effects. According to the billing regulations of PEPP, patients who remain in inpatient psychiatry at the turn of the year are assumed to be discharged on December 31 and readmitted on January 1 without subsequent case consolidation for accounting purposes. Thus, one case is technically split into two with shorter LOS, which may bias our results.

For the second subgroup analysis, we only considered the first hospital stay of each individual in the observation period. As Pletscher [17] suggests, marginal price effects are larger among moderately ill compared to severely ill patients. Assuming that patients who are readmitted to psychiatric hospitals are sicker than those who are not [40], our 2015 sample may be biased towards severe cases. One might argue that the model specification focussing on a short time window is not sufficiently granular to capture the effects of changes in per diem rates on the LOS, as a decrease in per diem payments might be over- or under-compensated by strategic discharges in other intervals of the LOS. Physicians with preferences for patient benefit and financial profits might redistribute resources across patients. This could affect the LOS in the population, but would not transform into significant changes of the probability of discharge in the time window considered in the baseline model specification described above. To investigate whether reimbursement incentives led to strategic discharge behaviour, which affected other parts along the LOS distribution, we conducted a discrete time duration analysis with time-varying coefficients for cost groups with a sufficient sample size (PA03B and PA04C). For a detailed description of the method, see appendix.

## Results

### Descriptive results

In 2014 and 2015, there were 12,627 cases for 10,402 individual patients at 82 hospitals that fulfilled the inclusion criteria. A total of 4957 cases were treated in 2014, while 7670 cases were treated in 2015. This difference can be explained by the fact that some of the hospitals opted for PEPP in the course of 2014 and applied the payment system all year in 2015. As we only considered cases billed according to PEPP, in those hospitals, fewer observations were made in 2014.

The patient populations in 2014 and 2015 exhibit quite similar characteristics (Table 2). The patients’ ages were 40.35 years in the intervention and 41.10 years in the comparison period. More women than men were treated in both periods (59% and 57% in 2014 and 2015, respectively). The distribution of observations on different cost groups is very similar in both periods. The costs group PA04C, which comprises cases with affective, neurotic, stress, somatoform or sleep disorders with an age below 65 years and without complications, accounts for more than half of the cases in both 2014 and 2015 (51%, respectively, 53%. Cost group PA03B (schizophrenia, schizotypal and delusional disorders, or other psychotic disorders, age < 65 years, without complications) accounts for 17% of all cases in both years. The patients are similarly distributed among the other cost groups in both years. The same is true for the distribution on ICD-10 F main diagnoses. Regarding mortality rates, 2% of the cases died within 360 days after discharge in both the intervention and control group. Compared to 2014, the average LOS was 0.66 days shorter in 2015 (Table 2).

**Table 2:** Summary statistics for PEPP admissions, 2014–2015

	2014		20115		Total	
	Mean	SD	Mean	SD	Mean	SD
Case-specific variables						
Length of stay (days)	34.45	32.35	33.79	32.40	34.05	32.38
Age (in years)	40.35	17.12	41.10	17.38	40.81	17.28
Female (%)	58.50	49.28	57.46	49.44	57.87	49.38
Discharge before MR declines (%)	35.00	47.70	35.12	47.74	35.08	47.74
Discharge 1 day before MR declines (%)	1.49	12.13	1.15	10.65	1.28	11.25
Discharge 1 day after MR declined (%)	1.29	11.29	1.08	10.35	1.16	10.73
Discharge 3 days before MR declines (%)	4.86	21.51	4.56	20.87	4.68	21.12
Discharge 3 days after MR declined (%)	3.97	19.54	4.13	19.91	4.07	19.76
Discharge 5 days before MR declines (%)	7.63	26.54	7.67	26.61	7.65	26.58
Discharge 5 days after MR declined (%)	7.24	25.92	7.12	25.72	7.17	25.80
Discharge 10 days before MR declines (%)	1.557	36.26	16.84	37.43	16.35	36.98
Discharge 10 days after MR declined (%)	13.60	34.28	13.94	34.64	13.80	34.50
Calendar month of admission	7.20	3.47	6.51	3.48	6.78	3.49
First admission in observation period (%)	89.07	31.21	78.04	41.40	82.37	38.11
360-day mortality (%)	1.65	12.76	1.89	13.62	1.80	13.29
Number of secondary diagnoses	2.34	2.52	2.36	2.57	2.35	2.55
PA03A (%)	2.58	15.86	2.50	15.62	2.53	15.72
PA03B (%)	17.31	37.84	16.96	37.53	17.10	37.65
PA04A (%)	1.59	12.52	1.77	13.20	1.70	12.93
PA04B (%)	11.14	31.46	11.19	31.52	11.17	31.50
PA04C (%)	51.08	49.99	52.83	49.92	52.14	49.96
PA14B (%)	6.56	24.75	7.11	25.69	6.89	25.33
PK04B (%)	6.78	25.14	5.70	23.18	6.12	23.97
PK10Z (%)	2.97	16.97	1.94	13.80	2.34	15.13
Main diagnosis F0 (%)	0.79	8.84	1.02	10.03	0.93	9.58
Main diagnosis F1 (%)	0.00	0.00	0.00	0.00	0.00	0.00
Main diagnosis F2 (%)	19.73	39.80	19.13	39.33	19.36	39.52
Main diagnosis F3 (%)	55.01	49.75	57.34	49.46	56.43	49.59
Main diagnosis F4 (%)	14.26	34.97	13.06	33.70	13.53	34.21
Main diagnosis F5 (%)	3.45	18.25	1.99	13.98	2.57	15.81
Main diagnosis F6 (%)	5.99	23.74	6.77	25.12	6.46	24.59
Main diagnosis F7 (%)	0.02	1.42	0.00	0.00	0.01	0.89
Main diagnosis F8 (%)	0.00	0.00	0.00	0.00	0.00	0.00
Main diagnosis F9 (%)	0.75	8.61	0.69	8.28	0.07	8.41
Hospital specific variables						
Cases per hospital	273.95	155.68	244.41	146.08	256.00	150.61
Experience with PEPP (days)	152.11	104.93	433.20	164.44	322.85	198.97
Cases in hospitals with PIA (%)	88.44	31.98	90.94	28.71	89.96	30.06
Cases in hospitals with day clinic (%)	82.55	37.96	86.22	34.47	84.78	35.92
Cases in hospitals with < 100 cases (%)	11.58	32.00	14.95	35.66	13.63	34.31
Inhabitants (hospital location)	612,889	1,041,101	512,600	912,193	551,974	966,061
*N*	4957		7670		12,627	

**Fig. 2:**
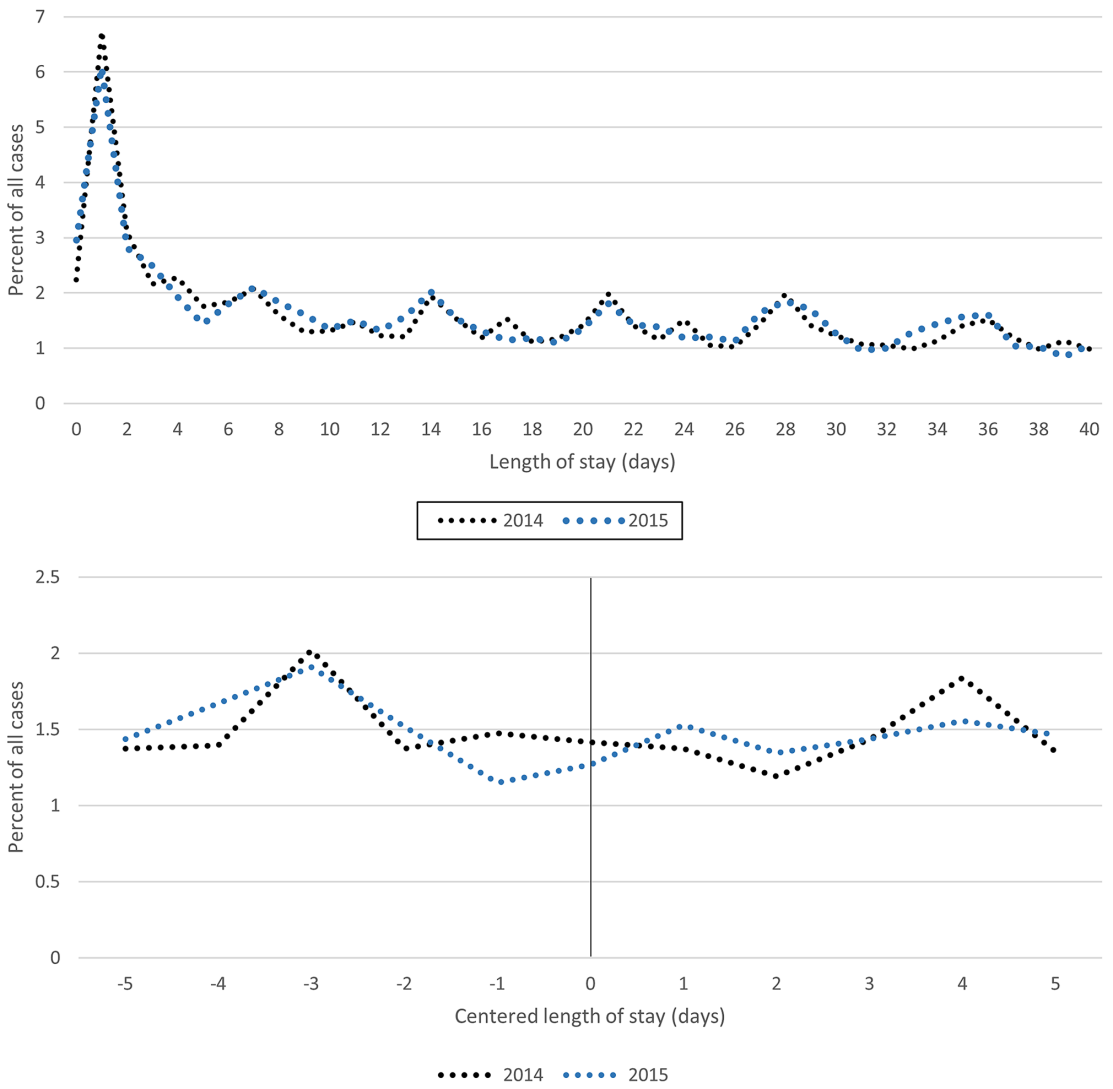
Observed density of LOS and centred LOS in 2014 and 2015

The observed density of LOS, which is depicted in Fig. 2, exhibits a similar pattern in both years. In 2014, 2.2% (6.7%) of cases were discharged on the same day (on the following day) of hospitalization. In 2015, this figure was 3.0% (6.1%). The proportion of cases discharged within the first 40 days was slightly higher in 2015 (66.8%) than in 2014 (65.8%). Only on some particular days of LOS (e.g. day 5 (1.8 vs. 1.4%), day 17 (1.5 vs. 1.1%)) or day 34 (1.1 vs. 1.4%)), small differences may be observed.

The observed density of the centred LOS, which is also shown in Fig. 2, allows us to conclude whether we can observe different patterns in discharge behaviour with and without discontinuous reimbursement based on descriptive statistics. Because LOS is centred at the threshold (LOS-*t*), we can compare discharge behaviour at the threshold over cost groups for the two years. A centred length of stay of zero indicates that a patient left the hospital on the first day that lower marginal reimbursement was realized in 2014. There is a slight peak in the relative number of admissions 3 days before the reduction in marginal revenue, while compared to 2015, fewer patients are discharged 2 days after the reduced tariff is applied. However, the two distributions do not reveal striking differences.

This finding is confirmed by the descriptive statistics shown in Table 2. The proportion of patients discharged within 1, 3 and 5 days before and after the threshold is approximately equal in both years.

### Regression results

The main regression results are displayed in Table 3. The first two columns show the estimated coefficients and standard errors for the regression model based on the entire set of observations. In the left two columns, regression results for the restricted sample, which only contains observations discharged within 3 or 5 days around the threshold, are displayed. The estimated coefficients for the “Treat” variable are insignificant on a 10% level for all regression specifications and samples.

**Table 3:** Basic regression results

*Dependent variables*	Full sample		Restricted sample	
	3 days	5 days	3 days	5 days
Variable of interest				
Treat	0.1625 (0.2878)	0.0565 (0.2275)	− 0.0405 (0.4503)	0.2694 (0.3321)
Case-specific variables				
Age	0.0415*** (0.0132)	0.0294*** (0.0102)	0.0548*** (0.0189)	0.0157 (0.0143)
Age^2^	– 0.0005*** (0.0002)	– 0.0004*** (0.0001)	– 0.0007*** (0.0002)	– 0.0003** (0.0002)
Female	– 0.1444 (0.0883)	– 0.1254* (0.0703)	– 0.1550 (0.1351)	– 0.0977 (0.1010)
Hospital-specific variables				
PEPP experience	– 0.0003 (0.0003)	– 0.0003 (0.0002)	– 0.0005 (0.0005)	– 0.0003 (0.0004)
PIA	– 0.0252 (0.2086)	– 0.0401 (0.1620)	– 0.1002 (0.3214)	0.1049 (0.2253)
Day clinic	– 0.2154* (0.1140)	– 0.1803* (0.0921)	– 0.1927 (0.1771)	– 0.2315* (0.1347)
Small hospital	0.0018 (0.1312)	0.0376 (0.1026)	0.0113 (0.1995)	0.098 (0.1501)
Urban	0.2297 (0.1417)	0.1758 (0.1108)	– 0.0761 (0.2179)	– 0.0245 (0.1540)
PIA * Treat	– 0.2253 (0.2848)	– 0.1793 (0.2257)	– 0.1203 (0.4476)	– 0.4615 (0.3306)
Comorbidities				
Elixhauser groups	Yes	Yes	Yes	Yes
PBM groups	Yes	Yes	Yes	Yes
*N*	12,627	12,627	1105	1871
Number of positive responses	591	966	591	966

Standard errors are in parenthesis.

*p < 0.10, **p < 0.05, ***p < 0.01

Patients’ age and squared age had a significant effect on the probability of being discharged within 3 days before the threshold compared to being discharged on another day of the hospital stay. For the regression model based on the restricted sample with a binary variable indicating whether a patient has been discharged within 5 days before the threshold, the effect of age is insignificant. The coefficient for being female is negative, but not significant at the 5% level in all considered regression models. The hospital-specific characteristics have no significant effect on the probability of being discharged before the threshold.

### Sensitivity analysis and robustness checks

To control for unobserved hospital characteristics, we estimated an alternative model specification with hospital fixed effects. The results are shown in Table 4, column (1).

**Table 4:** Robustness checks

	(1)				(2)				(3)			
	Fixed effects				Exclusion December and January				First case			
	Full sample		Restricted sample		Full sample		Restricted sample		Full sample		Restricted sample	
*Dependent variable*	3 days	5 days	3 days	5 days	3 days	5 days	3 days	5 days	3 days	5 days	3 days	5 days
*Variable of interest*												
Treat	0.0521 (0.0870)	− 0.0153 (0.0706)	0.0730 (0.1356)	− 0.0385 (0.1026)	0.1432 (0.3290)	0.0460 (0.2605)	− 0.2827 (0.5176)	0.1405 (0.3799)	0.1607 (0.3065)	0.0452 (0.2455)	0.0657 (0.4817)	0.2287 (0.3588)
*Controls*												
Socio-demographics	✔	✔	✔	✔	✔	✔	✔	✔	✔	✔	✔	✔
Hospital characteristics					✔	✔	✔	✔	✔	✔	✔	✔
Hospital fixed effects	✔	✔	✔	✔								
Elixhauser and PBM groups	✔	✔	✔	✔	✔	✔	✔	✔	✔	✔	✔	✔
*N*	12,627	12,627	1105	1871	10,269	10,269	888	1499	10,401	10,401	896	1510
Number of pos. responses	591	966	591	966	477	783	477	783	497	793	497	793

Standard errors are in parenthesis.

*p < 0.10, **p < 0.05, ***p < 0.01

In this scenario, standard errors decrease, indicating estimates are more precise. The signs of three of the estimated coefficients change. However, coefficients are close to zero and odds ratios are close to one, while none of them is significant on a 10% level. Hence, no evidence in favour of a treatment effect can be derived from estimates in this model specification.

The results for the subgroup analysis, where cases discharged in December and admitted in January were excluded, are shown in column (2). The estimated coefficients of the variable indicating discontinuous marginal reimbursement are insignificant on a 10% level, and the sign does not change compared to the baseline specification.

Furthermore, we estimated regression models using samples, where only the first admission of each insurant was included to avoid a bias to severe cases. The results are depicted in column (3) of Table 4. However, again, we see no significant effect on the probability of being discharged 3 or 5 days before the threshold for this subsample either.

To assess whether cuts in reimbursement led to changes in the probability of discharge at different intervals of the LOS distribution, we estimated a discrete time duration model with time-varying coefficients for the two cost groups with largest sample size. However, we did not find reliable evidence in favour of a time-varying treatment effect over LOS. The results are displayed in the appendix.

## Discussion

In this study, we investigate whether cuts in marginal reimbursement over LOS have an impact on the discharge behaviour of mental health-care hospitals in Germany. At a time where treatment costs for mental diseases are strongly increasing [41] and many European countries are implementing PPS for inpatient psychiatric facilities (e.g. the Netherlands and Switzerland), this study produces important policy-related findings. A key difference of our work compared to the existing literature is that we do not investigate the introduction of a PPS, but rather analyse changes in per diem rates in an implemented system. New reimbursement systems usually involve multiple systemic changes, i.e. with respect to the average level and the distribution of payment rates. For example, they often entail new or revised patient classification systems, which makes it difficult to compare patient groups. Thus, our analysis offers the opportunity to measure the effect of changes in marginal reimbursement only.

The results of this study indicate that, at least at an early stage of the system, marginal price incentives induced by discontinuities in hospital reimbursement schedules, i.e. changes in reimbursement between 16.3% and 31.8% do not result in strategic discharge behaviour of psychiatric inpatient hospitals in Germany. That is, we did not find significant differences in the probability of being discharged before a certain LOS threshold, neither did we find reliable evidence that the probability of discharge between the two groups differed in other intervals of the LOS distribution.

Previous studies yield mixed evidence concerning the effect of marginal reimbursement incentives on treatment duration for patients with psychiatric disorders. Norton et al. [39] examined a natural experiment in which hospitals switched from retrospective per diem to prospective per case payment, i.e. the marginal price per day decreases to zero, and hospitals bear the full marginal costs. Using data on severely mentally ill patients, the authors estimate both average and marginal price elasticities for inpatient psychiatric cases and test whether LOS declines after the introduction of per case PPS. They found that the marginal price elasticity is not significantly different from zero, which is in line with our findings.

Several studies address marginal reimbursement incentives induced by discontinuously increasing prospective payment. Douven et al. [18] found that mental health-care providers respond to discontinuously increasing reimbursement schedules by expanding the treatment duration.

Two recently published works examine the impact of the introduction of a discontinuous prospective reimbursement schedule for long-term care hospitals in the USA [19, 20]. Hospitals are reimbursed by per diem payments until a threshold LOS. If the threshold LOS is exceeded, they receive a large lump sum payment to cover all following days [20].

Both studies find that discharges increase substantially once the threshold is passed. Because they indicate that marginal reimbursement incentives induced by discontinuous reimbursement schedules influence the timing of discharge, the findings of the three studies contrast with ours.

An explanation might be the design of the considered payment systems (increasing vs. decreasing reimbursement schedules), which may differentially influence physicians’ decision-making. As Ellis and McGuire [5] propose, when deciding on treatment, physicians weigh patient benefit and hospital profit against each other. With discontinuously decreasing reimbursement rates, hospital profits may increase when the patient’s treatment duration is decreased. With discontinuously increasing reimbursement rates, physicians may be incentivized to enhance the treatment duration of a patient to increase hospital profit. When discharging relatively earlier as opposed to later does not affect patients’ benefit in the same way, physicians’ response to declining versus increasing marginal reimbursement over LOS may differ. Assuming that an early discharge is more harmful to patients than being discharged later than is optimal, this could explain why with declining rates, we do not find evidence in favour of strategic discharge behaviour, which is an important implication for policy makers. If the regulator aims to set incentives to expand treatment duration, increasing reimbursement schedules might be a useful instrument, while reductions in LOS might not be achieved by cuts in reimbursement over LOS.

Declining discontinuous reimbursement schedules and their impact on LOS have also been subject to analysis. Pletscher examines whether the introduction of a mixed reimbursement system led to changes in the timing of discharge in Switzerland [17]. His results provide evidence in favour of a marginal price effect on LOS in inpatient psychiatric facilities. However, the reduction in marginal revenue must be sufficiently large. Furthermore, he found the estimated effect to be smaller among patients with psychotic (ICD-10 F2) and affective disorders (ICD-10 F3).

This offers an explanation for differences between his and our findings, as our study population consists of 75% of these types of diagnoses. Additionally, he investigates marginal reimbursement incentives, which manifest at day 6 of LOS, while we focus on per diem payment reductions at the 20th or 25th percentile, i.e. days 10–22, depending on the cost group (see Table 1), of the LOS distribution. Evidence suggests that financial incentives may have a differential effect over LOS [8, 16]. Furthermore, Pletscher [17] investigated a mixed reimbursement system consisting of per diem rates and case payment depending on the timing of discharge, which might lead to stronger financial incentives compared to solely declining per diem reimbursement.

It should be noted that it is only profitable for hospitals to discharge patients earlier once marginal reimbursement reduces, if they either realize losses or face the opportunity to generate higher contributions by admitting new patients for whom higher per diem payments can be realized. If capacity utilization is low and hospitals have free beds, they would rather keep patients as long as they realize any profit and would not necessarily have an incentive to discharge earlier or to substitute patients. However, in the last 20 years, the number of psychiatric cases in Germany strongly increased, while outpatient capacities are still expandable. Average waiting times for elective inpatient treatment in psychiatric hospitals in Germany are reported to be one month [42].

The share of occupied beds as a proportion of all beds in German psychiatric hospitals was 93.3% in both years under consideration [43, 44], implying that capacity utilization was comparably high, and psychiatric hospitals and wards may have had an incentive to substitute patients. By comparison, the occupancy rate in general hospitals was 76.1% at the same time [43].

Our study also has several limitations. First, PEPP is not perfectly prospective. Payment varies with treatment, not just with diagnosis, and hospitals receive additional fees (“Zusatzentgelte”) for specific treatments, thus mitigating incentives to reduce costs. Furthermore, in the first years after the introduction of the PEPP system, i.e. from 2013 to 2016, hospitals were partly compensated if their realized revenues were below their negotiated budget. When their revenues exceeded the negotiated budget, providers had to repay some of those revenues, which may reduce reimbursement incentives. However, we assume that it did not eliminate them. Theory on hospital behaviour suggests that profits are part of hospitals’ objective function [45, 46] and even non-profit facilities pursue profit-increasing activities to finance their missions [47]. If hospitals put an emphasis on profits, there exists an inherent incentive to increase the ratio of revenues to costs irrespective of the budget, which is determined based on the expected volume of services. This volume will be reimbursed regardless of the actual costs incurred. If hospitals do not substantially deviate from negotiated budgets, higher contribution margins, i.e. the difference of marginal revenue and marginal costs, will lead to higher profits, such that marginal financial incentives are evident.

Second, the observation period during which hospitals were observed after the smoothing of changes in per diem rates over LOS is only one year. If changes in hospital behaviour occur with a time delay, our results do not capture this effect.

Third, one crucial assumption of our study is that the marginal costs do not discontinuously change at the threshold LOS. If they fall proportionally to per diem rates, revenue per additional inpatient stay remains stable; hence, there would be no incentive for hospitals to alter their discharge behaviour [48]. Although we are not able to test that assumption due to the lack of cost data, it seems plausible to suppose that costs of treatment do not abruptly change at the 20th–25th percentile of the LOS distribution.

Fourth, our results may not be generalizable to conditions with short or very long average LOS. Other studies suggest that financial incentives may have differential effects on discharge behaviour over LOS [8, 16]. However, as we provide evidence on marginal reimbursement incentives manifesting at an LOS of 10–22, depending on the cost group, we are only able to draw conclusions about this treatment duration.

Fifth, it should be noted that only hospitals that decided to opt for the PEPP system were part of our analysis. We thus enhance comparability between treatment and control group, but we do not control for self-selection.

Furthermore, it is important to point out that cases in our sample are not representative for all inpatient psychiatric cases. For example, patients with mental and behavioural disorders due to drug abuse or disorders of psychological development have been excluded. The vast majority of cases (74.74%) suffer from schizophrenia, schizotypal and delusional disorders, as well as affective disorders, which are severe mental illnesses [39]. As evidence suggests that the impact of marginal reimbursement incentives differs across diagnoses [17], the results of this study are potentially not generalizable across all mental diseases.

It should further be noted that the German hospital market is characterized by a comparatively high proportion of not-for-profit hospitals. As evidence suggests that such hospitals react less sensitively to financial incentives in terms of adjusting inpatient psychiatric LOS [49], this could also have an impact on the generalizability of our results to other countries.

Furthermore, we are unable to analyse the effect of marginal price incentives on the intensity of treatment, because hospitals might not only manipulate LOS but also reduce their efforts to treat patients due to reductions in per diem payments. Hence, future research should shed light on the effects of marginal price incentives in mental health care on treatment intensity and quality outcomes.

Although we are able to control for individual-specific characteristics, such as age and comorbid conditions, to some extent, consideration of individual fixed or random effects in the regression models would be desirable [39]. This was not possible here, as only a very small proportion of patients in our sample were hospitalized in both observation periods.

## Conclusion

As reimbursement systems for mental health-care providers are changing to per diem prospective payment systems, it is critical to understand the implications of marginal reimbursement incentives. Per diem systems with discontinuous reimbursement schedules, which have been recently introduced in several countries, may exhibit marginal incentives to reduce LOS.

This paper investigates the impact of a payment reform implemented in Germany, where such marginal price incentives were abolished. The analysis using administrative data from the largest German sickness fund for 12,627 patients and 82 hospitals indicates that the application of a discontinuous per diem reimbursement schedule with abrupt reductions in marginal reimbursement of between 16 and 32% did not alter discharge behaviour of mental health-care providers. Interest associations’ concerns that such a digressive payment scheme would lead to premature discharges of patients in an unstable mental health condition are not confirmed by our results.

Therefore, we show that if regulators aim to set incentives to decrease LOS, this might not be achieved by cuts in reimbursement over LOS—at least if these cuts occur at the 20–25th percentile of the LOS distribution.

## Electronic supplementary material

Below is the link to the electronic supplementary material.

Supplementary file1 (DOCX 65 kb)

## Data Availability

This study uses claims data from a German health insurance company that are subject to strict data protection rules according to SGB V. Therefore, the data cannot be made publicly accessible.
