# Supplementary material for: Do discontinuities in marginal reimbursement affect inpatient psychiatric care in Germany?
Source: Eur J Health Econ. 2020 Nov 9;22(1):101–14. doi: 10.1007/s10198-020-01241-5 (PMC7822769; doi:10.1007/s10198-020-01241-5)
Supplement: Supplementary file 1 — Supplementary file1 (DOCX 65 kb) [file 10198_2020_1241_MOESM1_ESM.docx]

## Appendix to “Do discontinuities in marginal reimbursement affect inpatient psychiatric care in Germany?”

### A. Supplementary Tables

| **Table A.1** Designation of cost groups (PEPPS) | |
| --- | --- |
| **PEPP** | **Designation** |
| PA03A | Schizophrenia, schizotypal and delusional disorders or other psychotic disorders, age > 64 years or with complicating constellation or with high therapy intensity or with mother/father/child setting |
| PA03B | Schizophrenia, schizotypal and delusional disorders or other psychotic disorders, age < 65 years, without complicating constellation |
| PA04A | Affective, neurotic, stress, somatoform and sleep disorders, age > 89 years or with complicated diagnosis and age > 64 years, or with complicated constellation or with high therapy intensity |
| PA04B | Affective, neurotic, stress, somatoform and sleep disorders, age < 90 years, without complicated constellation, with mother/father-child setting or with complicated diagnosis |
| PA04C | Affective, neurotic, stress, somatoform and sleep disorders, age < 65 years, without a complicated constellation, without a complicated constellation diagnosis |
| PA14B | Personality and behavioural disorders, eating disorders and other disorders, age < 65 years, without complicated constellation |
| PK04B | Affective, neurotic, stress, somatoform and sleep disorders, without complicated secondary diagnosis, without individual and micro-group support at great expense |
| PK10Z | Eating and feeding disorders |

| **Table A.2** Summary statistics for restricted sample of PEPP admissions (3 days around threshold) | | | | | | | | |
| --- | --- | --- | --- | --- | --- | --- | --- | --- |
|  | 2014 | |  | 2015 | |  | Total | |
|  | Mean | Std Dev |  | Mean | Std Dev |  | Mean | Std Dev |
| *Case specific variables* |  |  |  |  |  |  |  |  |
| Age (years) | 42.78 | 17.49 |  | 42.27 | 17.26 |  | 42.47 | 17.34 |
| Female (%) | 60.27 | 48.99 |  | 52.47 | 49.98 |  | 56.65 | 49.58 |
| Length of stay (days) | 16.09 | 3.21 |  | 16.17 | 3.15 |  | 16.14 | 3.18 |
| Discharge before MR declines (%) | 55.02 | 49.80 |  | 55.02 | 49.80 |  | 53.48 | 49.90 |
| Discharge 1 day before MR declines (%) | 16.89 | 37.51 |  | 13.19 | 33.87 |  | 14.66 | 35.39 |
| Discharge 1 day after MR declined (%) | 14.61 | 35.36 |  | 12.44 | 33.03 |  | 13.30 | 33.98 |
| Discharge 3 days before MR declines (%) | 55.02 | 49.80 |  | 52.47 | 49.98 |  | 53.48 | 49.90 |
| Discharge 3 days after MR declined (%) | 44.98 | 49.80 |  | 47.53 | 49.98 |  | 46.52 | 49.90 |
| Calendar month of admission | 6.98 | 3.49 |  | 6.45 | 3.52 |  | 6.66 | 3.52 |
| 360-day mortality (%) | 2.29 | 14.95 |  | 1.50 | 12.16 |  | 1.81 | 13.34 |
| Number of secondary diagnoses | 2.30 | 2.46 |  | 2.21 | 2.22 |  | 2.25 | 2.32 |
| PA03A (%) | 3.20 | 17.61 |  | 2.70 | 16.22 |  | 2.90 | 16.77 |
| PA03B (%) | 24.20 | 42.88 |  | 19.79 | 39.87 |  | 21.54 | 41.12 |
| PA04A (%) | 2.05 | 14.20 |  | 2.40 | 15.31 |  | 2.26 | 14.88 |
| PA04B (%) | 11.42 | 31.84 |  | 11.99 | 32.51 |  | 11.76 | 32.23 |
| PA04C (%) | 42.92 | 49.55 |  | 49.93 | 50.04 |  | 47.15 | 49.94 |
| PA14B (%) | 10.27 | 30.40 |  | 9.90 | 29.88 |  | 10.05 | 30.07 |
| PK04B (%) | 5.02 | 21.87 |  | 2.40 | 15.31 |  | 3.44 | 18.23 |
| PK10Z (%) | 0.91 | 9.52 |  | 0.90 | 9.45 |  | 0.90 | 9.47 |
| Main diagnosis F0 (%) | 0.68 | 8.26 |  | 1.80 | 13.30 |  | 1.36 | 11.58 |
| Main diagnosis F1 (%) | 0.00 | 0.00 |  | 0.00 | 0.00 |  | 0.00 | 0.00 |
| Main diagnosis F2 (%) | 27.63 | 44.77 |  | 21.89 | 41.38 |  | 24.16 | 42.83 |
| Main diagnosis F3 (%) | 47.49 | 49.99 |  | 54.72 | 49.81 |  | 51.86 | 49.99 |
| Main diagnosis F4 (%) | 13.01 | 33.68 |  | 10.64 | 30.86 |  | 11.58 | 32.02 |
| Main diagnosis F5 (%) | 1.60 | 12.55 |  | 0.90 | 9.45 |  | 1.18 | 10.79 |
| Main diagnosis F6 (%) | 9.59 | 18.48 |  | 9.30 | 29.06 |  | 9.41 | 29.21 |
| Main diagnosis F7 (%) | 0.00 | 0.00 |  | 0.00 | 0.00 |  | 0.00 | 0.00 |
| Main diagnosis F8 (%) | 0.00 | 0.00 |  | 0.00 | 0.00 |  | 0.00 | 0.00 |
| Main diagnosis F9 (%) | 0.00 | 0.00 |  | 0.75 | 8.63 |  | 0.45 | 6.71 |
| *Hospital specific variables* |  |  |  |  |  |  |  |  |
| Cases per hospital | 279.07 | 158.45 |  | 251.20 | 149.79 |  | 262.24 | 153.82 |
| Experience with PEPP (days) | 144.44 | 101.44 |  | 435.88 | 163.10 |  | 320.36 | 201.15 |
| Cases in hospitals with PIA (%) | 88.36 | 32.11 |  | 92.05 | 27.07 |  | 90.59 | 29.21 |
| Cases in hospitals with day clinic (%) | 81.74 | 38.68 |  | 83.96 | 36.73 |  | 83.08 | 37.51 |
| Cases in hospitals with < 100 cases (%) | 9.82 | 29.79 |  | 14.99 | 35.73 |  | 12.94 | 33.58 |
| Inhabitants (hospital location) | 704674 | 1151726 |  | 579983 | 1023574 |  | 629408 | 1077415 |
| N | 438 | |  | 667 | |  | 1 105 | |

| **Table A.3** Summary statistics for the restricted sample of PEPP admissions (5 days around threshold) | | | | | | | | |
| --- | --- | --- | --- | --- | --- | --- | --- | --- |
|  | 2014 | |  | 2015 | |  | Total | |
|  | Mean | Std Dev |  | Mean | Std Dev |  | Mean | Std Dev |
| *Case specific variables* |  |  |  |  |  |  |  |  |
| Age (years) | 42.34 | 16.99 |  | 42.05 | 17.17 |  | 42.16 | 17.09 |
| Female (%) | 57.94 | 49.40 |  | 55.11 | 49.76 |  | 56.23 | 49.62 |
| Length of stay (days) | 16.29 | 4.09 |  | 16.16 | 3.94 |  | 16.21 | 4.00 |
| Discharge before MR declines (%) | 51.29 | 50.02 |  | 51.85 | 49.99 |  | 51.63 | 49.99 |
| Discharge 1 day before MR declines (%) | 10.04 | 30.07 |  | 7.76 | 26.77 |  | 8.66 | 28.13 |
| Discharge 1 day after MR declined (%) | 8.68 | 28.18 |  | 7.32 | 26.06 |  | 7.86 | 26.91 |
| Discharge 3 days before MR declines (%) | 32.70 | 46.94 |  | 30.86 | 46.21 |  | 31.59 | 46.50 |
| Discharge 3 days after MR declined (%) | 26.73 | 44.29 |  | 27.95 | 44.91 |  | 27.47 | 44.65 |
| Discharge 5 days before MR declines (%) | 51.29 | 50.02 |  | 51.85 | 49.99 |  | 51.63 | 49.99 |
| Discharge 5 days after MR declined (%) | 48.71 | 50.02 |  | 48.15 | 49.99 |  | 48.37 | 49.99 |
| Calendar month of admission | 7.07 | 3.47 |  | 6.48 | 3.54 |  | 6.71 | 3.52 |
| 360-day mortality (%) | 2.31 | 15.02 |  | 1.59 | 12.50 |  | 1.87 | 13.55 |
| Number of secondary diagnoses | 2.38 | 2.45 |  | 2.23 | 2.36 |  | 2.29 | 2.40 |
| PA03A (%) | 3.39 | 18.11 |  | 2.56 | 15.79 |  | 2.89 | 16.75 |
| PA03B (%) | 21.57 | 41.16 |  | 19.58 | 39.70 |  | 20.36 | 40.28 |
| PA04A (%) | 1.90 | 13.66 |  | 2.38 | 15.25 |  | 2.19 | 14.64 |
| PA04B (%) | 10.99 | 21.30 |  | 10.93 | 31.22 |  | 10.96 | 31.24 |
| PA04C (%) | 46.13 | 49.88 |  | 50.26 | 50.02 |  | 48.64 | 49.99 |
| PA14B (%) | 10.58 | 30.78 |  | 10.32 | 30.43 |  | 10.42 | 30.56 |
| PK04B (%) | 4.61 | 20.99 |  | 3.09 | 17.30 |  | 3.69 | 18.85 |
| PK10Z (%) | 0.81 | 8.99 |  | 0.88 | 9.35 |  | 0.86 | 9.21 |
| Main diagnosis F0 (%) | 0.68 | 8.21 |  | 1.41 | 11.80 |  | 1.12 | 10.54 |
| Main diagnosis F1 (%) | 0.00 | 0.00 |  | 0.00 | 0.00 |  | 0.00 | 0.00 |
| Main diagnosis F2 (%) | 25.10 | 43.39 |  | 21.78 | 41.29 |  | 23.09 | 42.15 |
| Main diagnosis F3 (%) | 49.80 | 50.03 |  | 54.32 | 49.83 |  | 52.54 | 49.95 |
| Main diagnosis F4 (%) | 12.75 | 33.38 |  | 11.11 | 31.44 |  | 11.76 | 32.22 |
| Main diagnosis F5 (%) | 1.49 | 12.13 |  | 0.71 | 8.37 |  | 1.02 | 10.03 |
| Main diagnosis F6 (%) | 9.63 | 29.53 |  | 9.88 | 29.85 |  | 9.78 | 29.71 |
| Main diagnosis F7 (%) | 0.00 | 0.00 |  | 0.00 | 0.00 |  | 0.00 | 0.00 |
| Main diagnosis F8 (%) | 0.00 | 0.00 |  | 0.00 | 0.00 |  | 0.00 | 0.00 |
| Main diagnosis F9 (%) | 0.01 | 0.07 |  | 0.79 | 8.88 |  | 0.67 | 8.31 |
| *Hospital specific variables* |  |  |  |  |  |  |  |  |
| Cases per hospital | 274.10 | 156.76 |  | 247.88 | 148.78 |  | 258.21 | 152.47 |
| Experience with PEPP (days) | 146.84 | 101.24 |  | 434.23 | 161.89 |  | 321.02 | 199.10 |
| Cases in hospitals with PIA (%) | 88.87 | 31.47 |  | 91.09 | 28.50 |  | 90.22 | 29.71 |
| Cases in hospitals with day clinic (%) | 83.18 | 37.43 |  | 84.57 | 36.14 |  | 84.02 | 36.65 |
| Cases in hospitals with < 100 cases (%) | 10.99 | 31.30 |  | 15.08 | 35.80 |  | 13.47 | 34.15 |
| Inhabitants (hospital location) | 653160 | 1084020 |  | 574940 | 989987 |  | 605752 | 1028479 |
| N | 737 | |  | 1 134 | |  | 1 871 | |

| **Table A.4** Summary Statistics: Proportions Elixhauser and PBM groups | | | | | | |  |  |
| --- | --- | --- | --- | --- | --- | --- | --- | --- |
|  | 2014 | |  | 2015 | |  | Total | |
|  | Mean | Std Dev |  | Mean | Std Dev |  | Mean | Std Dev |
| Elixhauser Group 1 | 0.0135 | 0.1155 |  | 0.0090 | 0.0944 |  | 0.0108 | 0.1032 |
| Elixhauser Group 2 | 0.0373 | 0.1896 |  | 0.0154 | 0.1231 |  | 0.0240 | 0.1530 |
| Elixhauser Group 3 | 0.0059 | 0.0763 |  | 0.0043 | 0.0655 |  | 0.0049 | 0.0699 |
| Elixhauser Group 4 | 0.0048 | 0.0694 |  | 0.0017 | 0.0411 |  | 0.0029 | 0.0541 |
| Elixhauser Group 5 | 0.0089 | 0.0938 |  | 0.0048 | 0.0693 |  | 0.0064 | 0.0798 |
| Elixhauser Group 6 | 0.1198 | 0.3248 |  | 0.0597 | 0.2370 |  | 0.0833 | 0.2764 |
| Elixhauser Group 7 | 0.0113 | 0.1057 |  | 0.0057 | 0.0755 |  | 0.0079 | 0.0886 |
| Elixhauser Group 8 | 0.0095 | 0.0969 |  | 0.0059 | 0.0764 |  | 0.0073 | 0.0850 |
| Elixhauser Group 9 | 0.0321 | 0.1762 |  | 0.0180 | 0.1329 |  | 0.0235 | 0.1516 |
| Elixhauser Group 10 | 0.0299 | 0.1702 |  | 0.0172 | 0.1301 |  | 0.0222 | 0.1473 |
| Elixhauser Group 11 | 0.0317 | 0.1751 |  | 0.0166 | 0.1276 |  | 0.0225 | 0.1483 |
| Elixhauser Group 12 | 0.0111 | 0.1048 |  | 0.0056 | 0.0747 |  | 0.0078 | 0.0878 |
| Elixhauser Group 13 | 0.0512 | 0.2205 |  | 0.0316 | 0.1748 |  | 0.0393 | 0.1943 |
| Elixhauser Group 14 | 0.0176 | 0.1313 |  | 0.0083 | 0.0910 |  | 0.0120 | 0.1087 |
| Elixhauser Group 15 | 0.0178 | 0.1321 |  | 0.0087 | 0.0931 |  | 0.0123 | 0.1101 |
| Elixhauser Group 16 | 0.0008 | 0.0284 |  | 0.0003 | 0.0161 |  | 0.0005 | 0.0218 |
| Elixhauser Group 17 | 0.0016 | 0.0401 |  | 0.0007 | 0.0255 |  | 0.0010 | 0.0321 |
| Elixhauser Group 18 | 0.0014 | 0.0376 |  | 0.0007 | 0.0255 |  | 0.0010 | 0.0308 |
| Elixhauser Group 19 | 0.0024 | 0.0491 |  | 0.0012 | 0.0342 |  | 0.0017 | 0.0407 |
| Elixhauser Group 20 | 0.0131 | 0.1138 |  | 0.0061 | 0.0780 |  | 0.0089 | 0.0938 |
| Elixhauser Group 21 | 0.0063 | 0.0788 |  | 0.0046 | 0.0674 |  | 0.0052 | 0.0721 |
| Elixhauser Group 22 | 0.0101 | 0.0999 |  | 0.0048 | 0.0693 |  | 0.0069 | 0.0827 |
| Elixhauser Group 23 | 0.0468 | 0.2112 |  | 0.0236 | 0.1518 |  | 0.0327 | 0.1779 |
| Elixhauser Group 24 | 0.0155 | 0.1237 |  | 0.0068 | 0.0821 |  | 0.0102 | 0.1006 |
| Elixhauser Group 25 | 0.0587 | 0.2351 |  | 0.0219 | 0.1464 |  | 0.0364 | 0.1872 |
| Elixhauser Group 26 | 0.0014 | 0.0376 |  | 0.0010 | 0.0323 |  | 0.0012 | 0.0344 |
| Elixhauser Group 27 | 0.0121 | 0.1094 |  | 0.0043 | 0.0655 |  | 0.0074 | 0.0855 |
| Elixhauser Group 28 | 0.0847 | 0.2785 |  | 0.0536 | 0.2252 |  | 0.0658 | 0.2480 |
| Elixhauser Group 29 | 0.0982 | 0.2977 |  | 0.0531 | 0.2242 |  | 0.0708 | 0.2565 |
| Elixhauser Group 30 | 0.1553 | 0.3623 |  | 0.0945 | 0.2926 |  | 0.1184 | 0.3231 |
| Elixhauser Group 31 | 0.4166 | 0.4930 |  | 0.1995 | 0.3996 |  | 0.2847 | 0.4513 |
| PBM Group 1 | 0.0095 | 0.0969 |  | 0.0103 | 0.1010 |  | 0.0100 | 0.0994 |
| PBM Group 2 | 0.0381 | 0.1915 |  | 0.0404 | 0.1969 |  | 0.0395 | 0.1948 |
| PBM Group 3 | 0.1166 | 0.3210 |  | 0.1180 | 0.3226 |  | 0.1174 | 0.3220 |
| PBM Group 4 | 0.0335 | 0.1799 |  | 0.0365 | 0.1876 |  | 0.0353 | 0.1846 |
| PBM Group 5 | 0.0032 | 0.0567 |  | 0.0027 | 0.0523 |  | 0.0029 | 0.0541 |
| PBM Group 6 | 0.0002 | 0.0142 |  | 0.0003 | 0.0161 |  | 0.0002 | 0.0154 |
| PBM Group 7 | 0.0637 | 0.2443 |  | 0.0671 | 0.2503 |  | 0.0658 | 0.2480 |
| PBM Group 8 | 0.0637 | 0.2443 |  | 0.0681 | 0.2519 |  | 0.0664 | 0.2489 |
| PBM Group 9 | 0.0046 | 0.0680 |  | 0.0038 | 0.0614 |  | 0.0041 | 0.0640 |
| PBM Group 10 | 0.0176 | 0.1313 |  | 0.0153 | 0.1226 |  | 0.0162 | 0.1261 |
| PBM Group 11 | 0.0024 | 0.0491 |  | 0.0012 | 0.0342 |  | 0.0017 | 0.0407 |
| PBM Group 12 | 0.0014 | 0.0376 |  | 0.0008 | 0.0280 |  | 0.0010 | 0.0321 |
| PBM Group 13 | 0.0059 | 0.0763 |  | 0.0061 | 0.0780 |  | 0.0060 | 0.0774 |
| PBM Group 14 | 0.0115 | 0.1066 |  | 0.0086 | 0.0924 |  | 0.0097 | 0.0982 |
| PBM Group 15 | 0.1487 | 0.3558 |  | 0.1627 | 0.3691 |  | 0.1572 | 0.3640 |
| PBM Group 16 | 0.0444 | 0.2060 |  | 0.0405 | 0.1973 |  | 0.0421 | 0.2007 |
| PBM Group 17 | 0.0115 | 0.1066 |  | 0.0107 | 0.1028 |  | 0.0110 | 0.1043 |
| PBM Group 18 | 0.0024 | 0.0491 |  | 0.0033 | 0.0570 |  | 0.0029 | 0.0541 |
| PBM Group 19 | 0.2445 | 0.4298 |  | 0.2555 | 0.4362 |  | 0.2512 | 0.4337 |
| PBM Group 20 | 0.0034 | 0.0585 |  | 0.0037 | 0.0603 |  | 0.0036 | 0.0596 |
| PBM Group 21 | 0.1041 | 0.3054 |  | 0.1040 | 0.3053 |  | 0.1041 | 0.3054 |
| PBM Group 22 | 0.1333 | 0.3400 |  | 0.1411 | 0.3481 |  | 0.1380 | 0.3450 |
| PBM Group 23 | 0.0192 | 0.1371 |  | 0.0177 | 0.1320 |  | 0.0183 | 0.1340 |
| PBM Group 24 | 0.0087 | 0.0927 |  | 0.0060 | 0.0772 |  | 0.0070 | 0.0837 |
| PBM Group 25 | 0.2689 | 0.4434 |  | 0.2729 | 0.4455 |  | 0.2713 | 0.4447 |
| PBM Group 26 | 0.0500 | 0.2180 |  | 0.0471 | 0.2118 |  | 0.0482 | 0.2143 |
| PBM Group 27 | 0.5457 | 0.4980 |  | 0.5493 | 0.4976 |  | 0.5479 | 0.4977 |
| PBM Group 28 | 0.3702 | 0.4829 |  | 0.3748 | 0.4841 |  | 0.3730 | 0.4836 |
| PBM Group 29 | 0.0329 | 0.1783 |  | 0.0302 | 0.1713 |  | 0.0313 | 0.1741 |
| PBM Group 30 | 0.1846 | 0.3880 |  | 0.1664 | 0.3724 |  | 0.1735 | 0.3787 |
| PBM Group 31 | 0.0008 | 0.0284 |  | 0.0004 | 0.0198 |  | 0.0006 | 0.0235 |
| PBM Group 32 | 0.1523 | 0.3594 |  | 0.1593 | 0.3660 |  | 0.1566 | 0.3634 |
| N | 4 957 | |  | 7 670 | |  | 12 627 | |

### B. Discrete time duration model with a complementary log-log link funtion

In our main specification, we considered two intervals of time (3 days and 5 days) before cuts in per diem rates apply. One might argue that the model specification focussing on a short time window is not sufficiently granular to capture the effects of changes in per diem rates on the LOS, as a decrease in per diem payments might be over- or under-compensated by strategic discharges in other intervals of the LOS. This could transform into significant changes of the probability of discharge at other treatment durations than the ones considered in this paper.

In order to investigate whether such shifts in the LOS due to the reform of per diem rates could be observed; a discrete time duration model with time-varying coefficients is estimated. A change of the hazard curve over LOS intervals may provide evidence in favour of changes in discharge behaviour. As the LOS distribution as well as the threshold LOS, i.e. the treatment duration when reduced reimbursement applies, differ substantially between cost groups, an aggregate analysis is not meaningful. Therefore, this analysis is to be conducted separately for each group.

We employ a complementary log-log link function as the LOS is measured in discrete intervals and a complementary log-log model is the discrete time counterpart of continuous time proportional hazard models. Our approach is similar to that of Pletscher [17], who estimated a discrete time duration model with a difference-in-difference specification and time-varying coefficients. The complementary log-log link function specifies the dependence of the hazard on time and the independent variables. Unlike the logit link function, the complementary log-log is asymmetrical.

The model specification is described in what follows:

The hazard rate is defined as the probability that an event will occur at time $t$, given that it has not yet occurred, and can be estimated as the probability of discharge at time $t$ $P\left( Y_{t}=1 \right)$ employing a dataset with one observation per inpatient day. The binary dependent variable $Y_{t}$ indicates whether an inpatient is discharged at LOS $t$.

Just as in the baseline specification of the logistic regression model (see section ‘Empirical Strategy’), the binary control $T_{i}$ indicates whether an inpatient was treated when discontinuous per diem rates applied. As we aim to investigate whether this form of reimbursement incentivises changes in the discharge probability in other intervals of treatment duration additional to the ones examined in the baseline specification model, the treatment effect is allowed to vary over LOS. This is being implemented by an interaction term between the treatment indicator $T_{i}$ and interval dummies $I_{t}$, which indicate whether an inpatient day is observed later than day $t$ [17]. The interval indicators are determined by the percentiles of the LOS distribution (10^th^ percentile, 20^th^ percentile, the threshold LOS ($t - 3$ days, $t,$ $t + 3$ days), the median and the 75^th^ percentile). As an example: For PEPP PA03B the intervals indicate whether an inpatient day is observed later than day 2 $\left( I_{10} \right)$, day 7 ($I_{20})$, day 14 $(I_{t-3})$, day 17 $(I_{t})$, day 20 $(I_{t+3})$, day 24 $(I_{50})$ or day 46 $(I_{75})$. The treatment effect is allowed to vary over intervals of the hazard rate and the effect in one interval is compared to the effect in the previous.

To control for differences in the baseline probability of discharge, piecewise constant baseline hazards $B_{t}$ are incorporated in the model, which are estimated in daily intervals for the first week, in weekly intervals for the days 8-28, in two-weekly intervals for day 29-60 and one baseline hazard for observations thereafter.

To eliminate potential accounting effects, we identified cases discharged from hospital on December 31 and treat them as censored. Thereby, we avoid a potential bias of our estimations as according to the billing regulations of the PEPP system, patients who remain in inpatient treatment at the turn of the year are discharged for accounting purposes.

We further controlled for month-in-year effects $M_{i}$in the baseline specification and conducted an analysis employing vectors of specific hospital ($Hospital_{i}$) and case characteristics ($Case_{i}$) (see chapter ‘Empirical Strategy’ for details) and an additional analysis with hospital fixed effects ($H_{i})$. The corresponding model equations are as follows:

$$P\left( Y_{t}=1 \right)=1-exp(-\exp\left( g\left( . \right) \right)$$

$$g\left( \cdot\right)=\alpha_{t}B_{t}+\beta T_{i}+\gamma_{t}I_{t}+\delta_{t}I_{t}T_{i}+\eta M_{i} (1)$$

$$g\left( \cdot\right)=\alpha_{t}B_{t}+\beta T_{i}+\gamma_{t}I_{t}+\delta_{t}I_{t}T_{i}+\eta M_{i}+\theta Case_{i}+\lambda Hospital_{i} (2)$$

$$g\left( \cdot\right)=\alpha_{t}B_{t}+\beta T_{i}+\gamma_{t}I_{t}+\delta_{t}I_{t}T_{i}+\eta M_{i}+\mu H_{i} \left( 3 \right)$$

We estimated the equations for each different cost group, however the analyses led to non-convergence of the maximum likelihood estimator for all except the two with the largest sample sizes (PA03B: n = 2 159, inpatient days = 60 354; PA04C: n = 6 584, inpatient days = 189 788) such that we only report results for this subgroups.

The estimated coefficients of the analyses are displayed in Table B.1.

| **Table B.1** Regression results | | | | | | | |
| --- | --- | --- | --- | --- | --- | --- | --- |
|  | PA03B | | |  | PA04C | | |
|  | (1) | (2) | (3) |  | (1) | (2) | (3) |
| Treat | 0.1773 | 0.2406 | 0.1773 |  | 0.1629** | 0.3043** | 0.1699** |
|  | (0.1487) | (0.2571) | (0.1496) |  | (0.079) | (0.1283) | (0.0795) |
| Treat x I_10_ | -0.3447 | -0.3322 | -0.3438 |  | -0.019 | -0.0103 | -0.0176 |
|  | (0.2146) | (0.2147) | (0.2146) |  | (0.1316) | (0.1316) | (0.1316) |
| Treat x I_20_ | 0.0375 | 0.0472 | 0.0334 |  | -0.2229* | -0.2222* | -0.2236* |
|  | (0.1990) | (0.1991) | (0.1990) |  | (0.1285) | (0.1285) | (0.1285) |
| Treat x I_t-3_ | 0.0991 | 0.1078 | 0.0983 |  | 0.011 | 0.0089 | 0.0101 |
|  | (0.2196) | (0.2196) | (0.2196) |  | (0.1386) | (0.1386) | (0.1386) |
| Treat x I_t_ | 0.4273 | 0.4333 | 0.4356* |  | -0.0905 | -0.0914 | -0.0917 |
|  | (0.2626) | (0.2627) | (0.2627) |  | (0.1866) | (0.1866) | (0.1866) |
| Treat x I_t+3_ | -0.4392 | -0.438 | -0.4400* |  | 0.1353 | 0.1371 | 0.1362 |
|  | (0.2604) | (0.2604) | (0.2605) |  | (0.1639) | (0.1640) | (0.1639) |
| Treat x I_50_ | 0.1506 | 0.1575 | 0.1398 |  | -0.0476 | -0.0421 | -0.0487 |
|  | (0.1970) | (0.1972) | (0.1972) |  | (0.0914) | (0.0914) | (0.0914) |
| Treat x I_75_ | -0.1297 | -0.1074 | -0.1260 |  | -0.0695 | -0.068 | -0.083 |
|  | (0.1717) | (0.1721) | (0.1720) |  | (0.0934) | (0.0935) | (0.0936) |
| Month FE | ✓ | ✓ | ✓ |  | ✓ | ✓ | ✓ |
| Hospital covariates |  | ✓ |  |  |  | ✓ |  |
| Case covariates |  | ✓ |  |  |  | ✓ |  |
| Hospital FE |  |  | ✓ |  |  |  | ✓ |
| Inpatient days | 60 354 | 60 354 | 60 354 |  | 189 788 | 189 788 | 189 788 |
| AIC | 15916.6482 | 15954.4050 | 15953.1713 |  | 48773.9966 | 48747.4896 | 48496.0782 |
| Standard errors in parentheses. * p < 0.10, ** p < 0.05, *** p < 0.01 | | | | | | | |
|  |  |  |  |  |  |  |  |

Comparing the AIC of the models, for PEPP PA03B the one controlling for month fixed effects only yields the best model fit in terms of minimization of information loss. However, the estimated coefficients are quite robust to all model specifications. None of the estimated coefficients is significant on a 5%-level. Only the coefficients of the interaction $Treat x I_{t}$ (p-value = 0.0973) and $Treat x I_{t+3}$(p-value = 0.0911) in model (3) have a p-value slightly below 0.10, indicating changes in the treatment effect of the respective intervals compared to the previous. However, as we do not find a significant effect in the other specifications and one would have to be willing to accept a 10% error probability; this cannot be interpreted as evidence in favour of an effect induced by reimbursement incentives.

For cost group PA04C, model (3) has the highest relative quality according to the AIC.

The coefficient of the treatment indicator is the only one significant on a 5%-level. It gives an estimate of the treatment effect on the probability of discharge during the first two days of inpatient episodes. All but one coefficient of the interactions $Treat x I_{t}$, indicating changes in the treatment effect in an interval compared to the previous, are insignificant. Solely the coefficients of the covariate $Treat x I_{20}$ are significant, however, only on a 10%-level. The negative sign of the coefficient indicates a decrease in the treatment effect in this interval compared to earlier treatment durations.

Estimating three model specifications of the discrete time duration model for two different subgroups, we do not find robust evidence in favour of a treatment effect induced by cuts in marginal reimbursement. Thus, the results are in line with that of our main analysis.
